# Hydronephrosis Due to Bilateral Tubo-ovarian Abscess

**DOI:** 10.5811/cpcem.2019.10.44568

**Published:** 2020-01-06

**Authors:** Emily Fite, Jennifer Fitzgerald, Quinn Kistenfeger

**Affiliations:** * Saint Louis University School of Medicine, Saint Louis, Missouri; † Saint Louis University School of Medicine, Division of Emergency Medicine, Department of Surgery, Saint Louis, Missouri

## Abstract

A 27-year-old female presented to the emergency department with fevers, nausea, chills, and non-specific bilateral lower quadrant abdominal pain. A pregnancy test was negative. Computed tomography demonstrated moderate left hydronephrosis secondary to tubo-ovarian abscess (TOA). The abscess was so large it distorted local anatomy and compressed the ureters. She was prescribed merepenem and admitted for care by obstetrics/gynecology.

## CASE PRESENTATION

**Image 1:**
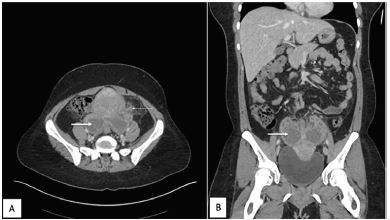
Computed tomography of abdomen and pelvis (A) axial view and (B) coronal view. Thick arrow: Bilateral adnexal multilocular septate cystic masses with enhancing septa and loss of normal ovarian parenchyma. Thin arrow: surrounding peritubal fat stranding.

**Image 2:**
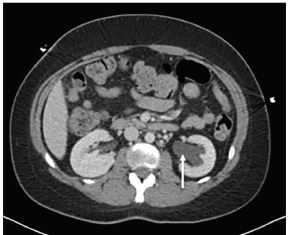
Computed tomography of the abdomen and pelvis in axial view demonstrating bilateral hydronephrosis, more prominent on the left (arrow) secondary to tubo-ovarian abscess.

A 27-year-old female presented to the emergency department with bilateral lower quadrant abdominal pain, fever, nausea, chills, and body aches. She stated she had been ill for three days and was getting worse. She was vomiting all oral intake and had new vaginal discharge. Upon examination, she was febrile to 101.1° Fahrenheit with a heart rate of 160 beats per minute. Her pregnancy test was negative. She had voluntary guarding and generalized tenderness on her abdominal exam while pelvic exam revealed cervical motion tenderness with copious vaginal discharge. Patient was given fluids and pain medication, and we obtained computed tomography (CT) of the abdomen and the pelvis with intravenous contrast (Images 1 and 2).

## DISCUSSION

This case demonstrates the complications that can occur when pelvic inflammatory disease goes untreated. Tubo-ovarian abscesses (TOA) can form from an ascending infection of the female genital tract leaking purulent discharge through the fallopian tube and forming a pus-filled mass encompassing the tube and/or ovary.^1^ In this case, the abscess was so large that it distorted local anatomy and compressed the ureters, causing hydronephrosis. Ultrasonography or CT can be used to evaluate nonspecific symptoms and look for specific complications associated with pelvic inflammatory disease.^2^ The CT demonstrated findings consistent with the presence of a TOA with hydronephrosis (Image 2).

First-line treatment of TOA with broad-spectrum antibiotics should begin immediately after blood cultures are taken.^1^ Treatment with antibiotics has been shown to be effective in many patients, but recurrence is likely. Surgical intervention may be considered if it is a recurrence, nonresponsive to antibiotics, or if rupture occurs.^1^,^3^ Minimally invasive measures should be considered, especially in women of childbearing age, to avoid causing infertility.^3^ Our patient was prescribed meropenem and she was admitted for care by her obstetrician-gynecologist.

**CPC-EM Capsule**

What do we already know about this clinical entity?

Untreated tubo-ovarian abscess (TOA) can lead to abscess rupture, sepsis, and infection of nearby organs.

What is the major impact of the image(s)?

In our case, the patient had a TOA so large that it put pressure on the ureters, causing bilateral hydronephrosis.

How might this improve emergency medicine practice?

TOA is a less common diagnosis. Treatment should begin promptly with broad-spectrum antibiotics and may require surgical intervention to prevent abscess rupture and sepsis.
